# Functional diversification of oxalate decarboxylases in terms of enzymatic activity, morphosporogenesis, stress regulation and virulence in *Colletotrichum siamense*

**DOI:** 10.3389/fmicb.2025.1547950

**Published:** 2025-02-28

**Authors:** Yanyun Lv, Yu Liu, Yuqing Lin, Huiying Zheng, Jingting Yan, Yu Zhang, Weiguo Miao, Wei Wu, Chunhua Lin

**Affiliations:** Sanya Institute of Breeding and Multiplication / Key Laboratory of Green Prevention and Control of Tropical Plant Diseases and Pests (Ministry of Education) / School of Tropical Agriculture and Forestry, Hainan University, Haikou, China

**Keywords:** *Colletotrichum siamense*, oxalate decarboxylase, morphosporogenesis, stress homeostasis, fungicide sensitivity, virulence

## Abstract

Oxalate decarboxylase (OxdC) is an enzyme that degrades oxalic acid and may affect the virulence of necrotrophic fungal pathogens that rely on oxalic acid as a pathogenicity factor. However, the biological function of OxdCs in hemibiotropic fungi is still unknown. Our previous studies revealed four OxdC-encoding genes in the whole genome, with *CsOxdC3* playing important roles in morphosporogenesis, fungicide resistance and virulence in *Colletotrichum siamense*. Here, we systematically analyzed the biological functions of four oxalate decarboxylase genes in *C. siamense* via a loss-of-function method. The results revealed *CsOxdC1*, *CsOxdC2*, and *CsOxdC4* played major roles in degrading oxalic acid in *C. siamense*, whereas *CsOxdC3* did not. All four *CsOxdCs* positively modulated morphosporogenesis, including vegetative growth, conidial size, conidial germination rate and the appressorium formation rate, to different extents. In particular, the *CsOxdC3* deletion mutant failed to form appressoria. The four *OxdC* gene deletion mutants had different responses to Mn^2+^, Cu^2+^, and multiple fungicides. Among them, *CsOxdC2* and *CsOxdC4* exhibited positive roles in resistance to Mn^2+^ and Cu^2+^ stresses; *CsOxdC1* played a slightly positive role in *C. siamense* resistance to azole fungicides; and *CsOxdC3* had a significantly positive role in regulating the sensitivity of *C. siamense* to multiple fungicides, including pyrrole and azole, but not *CsOxdC2* and *CsOxdC4*. Furthermore, compared with the wild-type strain, Δ*CsOxdC2* and Δ*CsOxdC3*, but not Δ*CsOxdC1* and Δ*CsOxdC4*, displayed significantly reduced virulence. In conclusion, our data indicated that *CsOxdCs* exerted diverse functions in morphogenesis, stress homeostasis, fungicide resistance, and virulence in *C. siamense*. This study provides insights into the biological function of *OxdCs* in the hemibiotrophic fungus *C. siamense*.

## 1 Introduction

*Colletotrichum* is one of the most common and important genera of filamentous fungal plant pathogens, comprising nine core clades with 15 major species complexes and 252 species (Khodadadi et al., 2023). Species of *Colletotrichum* employ diverse strategies for invading host tissue, ranging from intracellular hemibiotrophy to subcuticular intramural necrotrophy (Perfect et al., 1999). *Colletotrichum* spp. can cause anthracnose spots and blights in various economically important crops, especially fruits, vegetables and ornamentals (Dean et al., 2012). *C. siamense*, a member of the *C. gloeosporioides* species complex, is the dominant pathogen species in rubber tree, mango, litchi, and other tropical crops in the field (Liu et al., 2018; Ling et al., 2019; Qin et al., 2019). *Colletotrichum* has a long and distinguished history as a model pathogen for fundamental, biochemical, physiological and genetic studies (Dean et al., 2012). In recent years, although some functional genes of *Colletotrichum* have been characterized, additional genes related to pathogenicity and drug resistance need to be discovered to understand the pathogenesis of *Colletotrichum* and control the disease.

Oxalic acid (OA) is a natural organic acid with a low molecular weight and is an important metabolic product that is widely present in plants, animals, and microorganisms (Dutton and Evans, 1996; Grąz, 2024). It has strong acidity, reductivity, and the ability to chelate calcium (Dutton and Evans, 1996). Fungi can synthesize and secrete OA to maintain a suitable pH in their living environment (Dutton and Evans, 1996). OA is remarkably multifunctional in fungi and plays significant biological and pathological roles in the life cycle and infection processes, particularly in necrotrophic fungi. It can acidify the host tissue environment, participate in cell wall degradation, and induce reactive oxygen species production (Rollins and Dickman, 2001; Kim et al., 2008; Williams et al., 2011; Tian et al., 2021).

OA can be degraded by enzymes via decarboxylation or oxidation (Grąz, 2024). The enzymes involved in the biological degradation of OA in the biosphere include oxalyl-CoA decarboxylase, oxalate oxidase, and oxalate decarboxylase (Kumar et al., 2019). Among these enzymes, oxalate decarboxylase (OxdC), which is a manganese-dependent enzyme that can directly degrade OA into formic acid and CO_2_ without additional cofactors, is widely present in bacteria and fungi (Kumar et al., 2019). OxdCs belong to the bicupin protein family, which is widely involved in various life processes, including growth, development, and responses to environmental stress (Kathiara et al., 2000; Kesarwani et al., 2000; Chowdhury et al., 2024). Owing to the high specificity of OxdCs for their substrates and the high efficiency of their enzymatic reactions, some OxdCs have been extensively studied and successfully applied in agriculture, food, and other fields. In agricultural fields, OxdCs are often utilized for plant disease control. For example, tobacco, tomato, and lettuce harboring the *OxdC* genes from *Trametes versicolor*, *Collybia velutipes*, and *Flammulina* sp. have been shown to effectively defend against *Sclerotinia* infections (Kesarwani et al., 2000; Dias et al., 2006; Walz et al., 2008). Transgenic tomato expressing an *OxdC* gene from *F. velutipes* increased survival to *Moniliophthora perniciosa* (Pereira et al., 2022). Transgenic rice expressing an *OxdC* gene from *Bacillus subtilis* presented increased resistance to rice blast and rice sheath blight (Qi et al., 2017). Most studies on OxdCs focus on their enzyme activity and potential applications. However, the biological function of *OxdCs* in fungi has not been well characterized.

Some *OxdCs* have been characterized in plant pathogenic fungi, including *S. sclerotiorum*, *T. ochracea*, *T. versicolor*, *Postia placenta*, *Gleophylum trabeum*, *Serpula lacrymans*, and *Coniothyrium minitans* (Micales, 1997; Mäkelä et al., 2002; Grąz et al., 2011; Hastrup et al., 2012; Liang et al., 2015a; Liang et al., 2015b). Many studies have shown that multiple *OxdCs* exist, with diverse expression patterns and functions across different fungi. Currently, three expression patterns of oxalate decarboxylase genes have been identified: (1) inducible by OA and pH, such as the *OxdC* genes in *C. velutipes* and *Aspergillus niger* (Azam et al., 2002); (2) inducible solely by oxalate anions, as observed in the *OxdC* of the brown rot fungus (*P. placenta*) (Micales, 1997); and (3) not inducible by pH or OA, as observed in *S. sclerotiorum* with its *Ss-odc1* and *Ss-odc2* genes (Liang et al., 2015b). Different gene expression patterns suggest that *OxdCs* may have various functions. The biological functions of different *OxdCs* vary within the same fungi. For example, two *OxdC* genes have been identified in *S. sclerotiorum*, with only *Ss-Oxdc2* playing a role in oxalate degradation (Kabbage et al., 2013). In *C. minitans*, three *OxdC*s were analyzed, which revealed the involvement of *CmOxdC1* and *CmOxdC3* in OA degradation and their parasitic ability of *C. minitans* against host fungi; in contrast, *CmOxdC2* did not play a role in this process (Zeng et al., 2014; Xu et al., 2022). The OxdCs involved in plant pathogenicity are mainly necrotrophic fungi. Little is known about the biological function of OxdCs in hemibiotrophic or biotrophic fungi.

In our previous study, we identified four OxdC-encoding genes in the whole genome of *C. siamense*. We characterized the function of *CsOxdC3*, which interacts with CsPbs2 (a mitogen-activated protein kinase kinase) and is involved in morphogenesis, stress homeostasis, fungicide resistance, and virulence in *C. siamense* (Lu et al., 2024). In this study, we further investigated the functions of three other oxalate decarboxylase genes in *C. siamense* by comparing the phenotypes of the gene deletion mutants and wild-type strains. We found that all four *CsOxdCs* were involved in the growth and development of *C. siamense*, with differential regulation observed in terms of spore germination, sporulation, and appressorium formation. *CsOxdCs* were induced to varying degrees by OA and exhibited different levels of tolerance to OA. Enzyme activity assays revealed that *CsOxdC1*, *CsOxdC2*, and *CsOxdC4* possessed oxalate decarboxylase activity, whereas *CsOxdC3* had the weakest oxalate degradation ability. Additionally, the four *CsOxdCs* presented diverse responses to stress homeostasis, fungicide resistance, and virulence. Our research on the oxalate decarboxylase family in *C. siamense*, covering aspects such as morphogenesis, stress homeostasis, fungicide resistance, and virulence, indicates that *CsOxdCs* have diverse functions and may operate through different pathways in *C. siamense*.

## 2 Materials and methods

### 2.1 Fungal strains and culture conditions

The HN08 strain *C. siamense* from rubber trees was used as the wild-type (WT) strain. In this study, HN08 served as the starting strain to construct the gene deletion mutants Δ*CsOxdC1*, Δ*CsOxdC2*, and Δ*CsOxdC4* and the complementary mutants CΔ*CsOxdC1*, CΔ*CsOxdC2*, and CΔ*CsOxdC4*. The construction of Δ*CsOxdC3* and CΔ*CsOxdC3* (equivalent to Δ*CsOxdC3/CsOxdC3*) has been described in our previous study (Lu et al., 2024). The methods for constructing mutants and complementary strains have been detailed in a previous study (Liu et al., 2023; Lu et al., 2024). The gene deletion diagram and primers for PCR conformation are listed in Supplementary Figure S1. For spore collection, the strains were grown on potato dextrose agar (PDA: 20 g/L potato, 20 g/L glucose, and 18 g/L agar) in Petri dishes for 3 days, after which the hyphae were scraped off and inoculated into liquid potato dextrose medium (PD, PDA without agar) with shaking at 150 rpm and 28°C for 3–5 days, after which large numbers of spores were harvested with ddH_2_O for preparation of the spore suspension. DNA, RNA and total proteins were extracted from mycelial strains cultured in liquid complete medium (CM: 0.6% yeast extract, 0.1% casein hydrolysate, and 1% sucrose) with shaking at 150 rpm and 28°C for 3–5 days.

### 2.2 Gene expression analysis of *CsOxdCs*

The expression of the *CsOxdC* genes was analyzed via qRT–PCR. The final concentrations of OA were 0 and 6 mM in complete medium. HN08, *∆CsOxdCs* and C*∆CsOxdCs* were cultured in this medium for 2–3 days. Total RNA was extracted using the SteadyPure Plant RNA Extraction Kit (Accurate, China), and the RNA was reverse-transcribed into cDNA using the EvoM-MLV RT Mix Kit with gDNA Clean for qRT-PCR (Accurate, China). Two-step qRT–PCR (TOLOBIO, China) was used to compare the expression levels of the *CsOxdC* genes after 0 and 6 mM OA treatment. All primers used in this study are listed in Supplementary Table S1.

### 2.3 Assaying the tolerance of *Colletotrichum siamense* to OA

A total of 10 μL of the spore suspensions of HN08, *∆CsOxdCs* and C∆*CsOxdCs* at a concentration of 10^5^ conidia/mL were inoculated onto CM plates containing 0, 3, 6, 12, and 24 mM OA. Bromophenol blue was added to the media at 0.001% (w/v) as a pH indicator to monitor the pH changes caused by fungal growth. After incubation in the dark at 28°C, the colony diameter was measured, and the color of the media was observed and photographed after 5 days. The experiment was repeated three times. The inhibition rate was calculated using the following formula: inhibition rate (%) = [colony diameter (control group - treatment group)]/[colony diameter (control group)] × 100.

### 2.4 Determination of OA degradation ability

Spore suspensions of HN08, ∆*CsOxdCs*, and C∆*CsOxdCs* at the same concentration were inoculated into PD medium containing 12 mM OA. The OA contents in the medium at 0, 12 and 24 h were detected and recorded using an OA content detection kit (BOXNIO, China).

### 2.5 Oxalate decarboxylase activity assay

Spore suspensions of HN08 and ∆*CsOxdCs* at the same concentration were cultured in PD medium, and total protein was extracted using a Protein Extraction Kit (BestBio, China). The level of *OxdC* activity in *C. siamense* was detected using a colorimetry assay in accordance with the instructions supplied with the Oxalate Decarboxylase Activity Assay Kit (Mmbio, China). Briefly, *OxdC* activity was determined by generating a colorimetric product with absorbance at 450 nm (A450), proportional to the enzymatic activity present. One unit of oxalate decarboxylase was the amount of enzyme required to generate 1.0 mM formate per minute at pH 5 and 37°C.

### 2.6 Phenotype analysis

A total of 10 μL of the spore suspensions of HN08, *∆CsOxdCs* and C∆*CsOxdCs* at a concentration of 10^5^ conidia/mL were inoculated onto CM plates. The growth rate of the individual colonies was assessed 5 days postinfection (dpi) at 28°C. To measure conidial germination, the number of spores and appressorial formation, 10 μL of 10^5^ conidia/mL spore suspension preparations was dropped onto hydrophobic slides and incubated at 28°C according to a previous study (Song et al., 2022). The rate of spore germination at 0, 2, 4, 6 and 8 h and the rate of appressorium formation at 12 h post inoculation (hpi) was recorded. At least three hundred spores were tested, and three independent experiments were performed.

The stress responses of the HN08, Δ*CsOxdCs*, and CΔ*CsOxdCs* strains were examined by inoculating 10 μL of the 10^5^ conidia/mL conidial suspension from the gene deletion mutants, WT (HN08), and complementation strains on CM media supplemented with different chemical materials, including 10 mM Mn^2+^, 6 mM Cu^2+^, 1.0 μg/mL fludioxonil, 0.1 μg/mL fenpiclonil, 0.5 μg/mL tebuconazole and 0.01 μg/mL prochloraz, and culturing the samples at 28°C. The colony diameter was measured, and the colonies were photographed at 5 dpi. The growth inhibition rate was calculated using the following formula: inhibition rate (%) = [colony diameter (control group - treatment group)]/[colony diameter (control group)] × 100.

### 2.7 Pathogenicity assessment assays

Pathogenicity assessment was performed via drop inoculation of intact and injured tender leaves that had been detached from rubber trees with 10 μL of spore suspension (1 × 10^5^ conidia/mL) prepared with spores obtained from the individual strains. Three biological replicates were assayed, and 30 leaves were inoculated for each treatment. The diseased lesions were measured and photographed at 5 dpi.

## 3 Results

### 3.1 Identification and characteristics of *CsOxdCs* in *Colletotrichum siamense*

**Figure 1:**
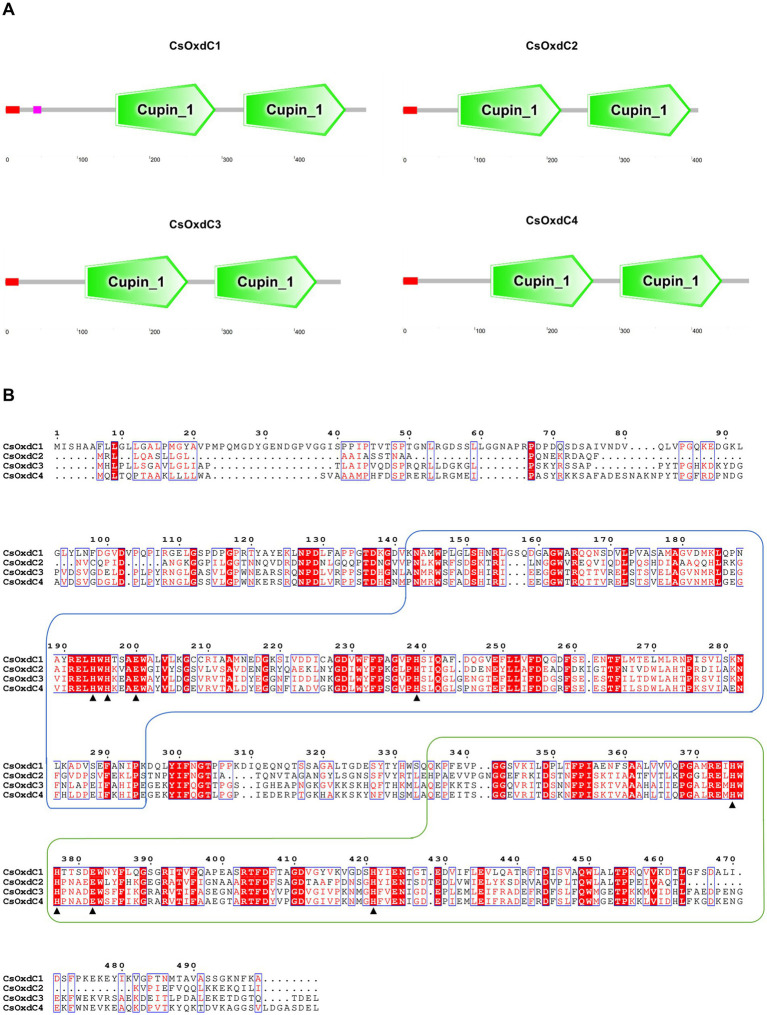
Protein domain and amino acid sequence analysis of the four members of *CsOxdCs* family in *C. siamense*. **(A)** SMART analysis of the four *CsOxdC* proteins. **(B)** The amino acid sequence alignment of four *CsOxdC* proteins. The two wireframes are the Cupin_1 domain; the red area is the conserved motif; ▲indicate amino acid residues for Mn^2+^-binding site. The sequences were aligned with Clustal X and analyzed using Espript3.0.

In our previous study, we identified four oxalate decarboxylase coding genes in *C. siamense* and characterized the biological function of the *CsOxdC3* gene among them (Lu et al., 2024). Here, we cloned and analyzed the remaining three oxalate decarboxylase genes in *C. siamense*. Sequence analyses revealed that the *CsOxdC1* gene had a DNA size of 1750 bp and contained 4 introns and 5 exons encoding 499 amino acids. The *CsOxdC2* gene was 1,568 bp in length and contained 7 introns and 8 exons, encoding 409 amino acids in total. The *CsOxdC4* gene consisted of 1,684 bp with 2 introns, encoding a total of 479 amino acids. SMART analysis revealed that all four *CsOxdC* proteins had two Cupin_1 domains containing Mn^2+^-binding sites and a signal peptide at the N-terminus (Figure 1). *CsOxdC1* also contained a low-complexity domain sequence (Figure 1A). Comparative analysis of the amino acid sequences revealed that *CsOxdC1* and *CsOxdC2* had low homology (less than 40%) with the other proteins, whereas *CsOxdC3* and *CsOxdC4* shared 76.41% homology (Figure 1B). This finding was consistent with previous phylogenetic analyses, which grouped OxdCs from fungi into five clades (A to E). *CsOxdC3* and *CsOxdC4* belonged to Clade A, *CsOxdC1* belonged to Clade D, and *CsOxdC2* belonged to Clade E in *C. siamense* (Lu et al., 2024).

### 3.2 *CsOxdC1*, *CsOxdC2* and *CsOxdC4* show oxalate decarboxylase enzyme activity in *Colletotrichum siamense*

**Figure 2:**
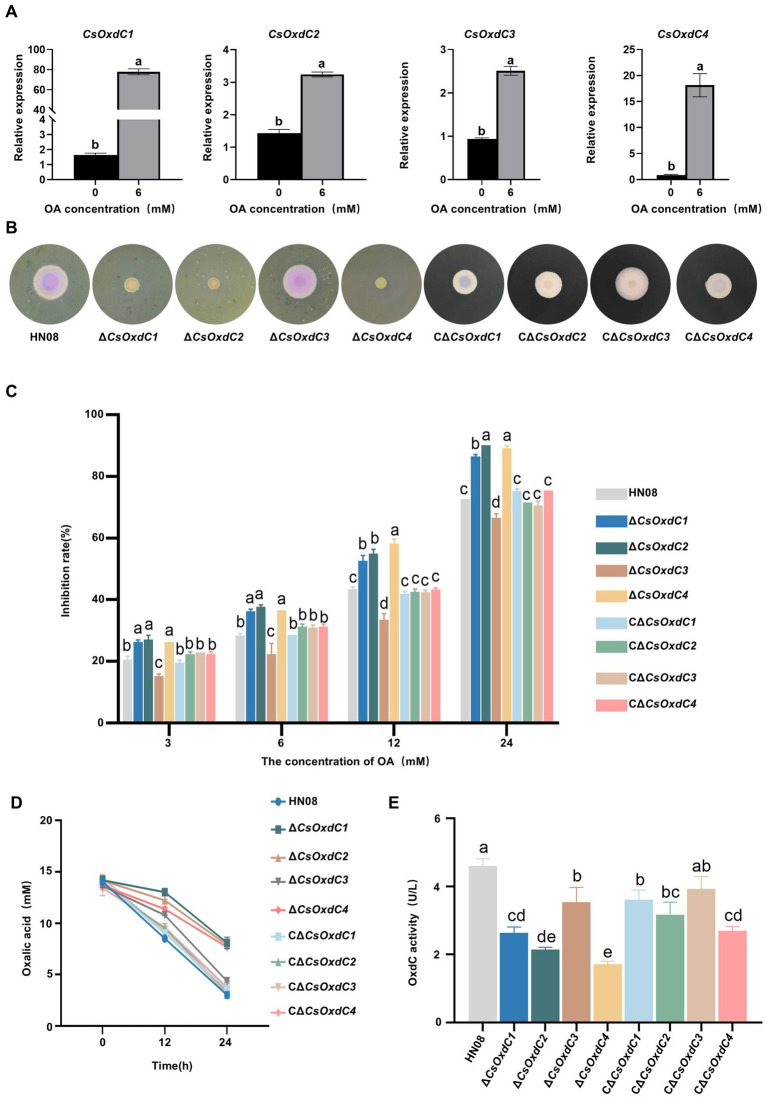
Gene expression and oxalate decarboxylase activities of the four *CsOxdC* members in *C. siamense*. **(A)** Effect of OA on gene expression of *CsOxdCs* family in *C. siamense*. **(B)** Colony size and medium color of the cultures of the tested strains on CM supplemented with bromophenol blue and 24 mM OA. **(C)** Growth inhibition rate of the tested strains under different concentrations of OA stress. **(D)** Content of OA in medium of the tested strains cultured at 0, 12 and 24 hpi. **(E)** The oxalate decarboxylase activities of the tested strains after being cultured in PD medium for 2 dpi. Different letters indicate significant differences (*p* < 0.05, according to one-way ANOVA and Duncan’s test), and the error bars represent the standard deviations.

Considering that OxdCs are enzymes that can directly degrade OA into formic acid and CO_2_ without additional cofactors (Kumar et al., 2019), we investigated whether these four OxdCs had oxalate decarboxylase enzyme activity in *C. siamense*. First, we evaluated whether the expression of the four *CsOxdC* genes was induced by 6 mM OA (Figure 2A). The results showed that the expression levels of the four *CsOxdC* genes were significantly elevated compared with the control, with *CsOxdC1* expression reaching a 76.38-fold increase, *CsOxdC2* expression reaching a 3.24-fold increase, *CsOxdC3* expression reaching a 2.51-fold increase, and *CsOxdC4* expression reaching a 18.13-fold increase (Figure 2A). These results demonstrated that the expression of the four *CsOxdC* genes was upregulated by OA induction, with varying degrees of upregulation among the different genes.

The *CsOxdCs*-deficient strains (Δ*CsOxdC1*, Δ*CsOxdC2*, Δ*CsOxdC3*, and Δ*CsOxdC4,* collectively named Δ*CsOxdCs*) were subsequently constructed by replacing each *CsOxdCs* gene with the *ILV1* gene (the sulfonylurea resistant gene) (Sweigard et al., 1997), which was confirmed via PCR amplification and sequencing. The complementary strains CΔ*CsOxdCs* (CΔ*CsOxdC1*, CΔ*CsOxdC2*, CΔ*CsOxdC3*, and CΔ*CsOxdC4,* also named CΔ*CsOxdCs*) were also constructed and identified by PCR confirmation (Supplementary Figure S1). The effects of *CsOxdCs* on tolerance to OA were tested by growing the Δ*CsOxdCs* mutants, *C*Δ*CsOxdCs* and WT strains on CM supplemented with OA at five concentrations (0, 3, 6, 12 and 24 mM). Since medium containing bromothymol blue acts as a pH indicator, changing color from blue to yellow upon the addition of OA (Zeng et al., 2014), we compared the intensity of the colony color shift between the four mutants and the WT at different OA concentrations (Figure 2B and Supplementary Figure S2). Notably, the color of the media colonized by Δ*CsOxdC1*, Δ*CsOxdC2*, and Δ*CsOxdC4* remained yellow, whereas that colonized by the WT and Δ*CsOxdC3* turned pink at 24 mM, indicating that Δ*CsOxdC1*, Δ*CsOxdC2*, and Δ*CsOxdC4* have lost the ability to degrade OA to some extent compared with WT and Δ*CsOxdC3*. Although the color of the complementation mutants CΔ*CsOxdCs* did not completely match that of the WT, which may be due to the reintroduced genes only partially restored the phenotype (Figure 2B). The growth inhibition rates of the Δ*CsOxdC1*, Δ*CsOxdC2*, and Δ*CsOxdC4* mutants were significantly greater than those of the WT strain under various concentrations of OA. In contrast, the growth inhibition rates of Δ*CsOxdC3* were significantly lower than those of the WT (Figures 2B,C). Additionally, the growth rates of the complementation mutants CΔ*CsOxdCs* did not significantly differ from those of the WT (Figure 2C). Both the colony color and growth inhibition data suggested that the primary OA-degrading genes of this family were *CsOxdC1*, *CsOxdC2* and *CsOxdC4* but not *CsOxdC3* in *C. siamense*.

Furthermore, a reduction in the OA concentration was observed in the culture solutions of the four mutants and WT initially 12 mM OA added (Figure 2D). The reducing levels of Δ*CsOxdC1*, Δ*CsOxdC2*, and Δ*CsOxdC4* were similar. The most significant decrease in OA was observed in the WT and Δ*CsOxdC3* strains at 24 h, indicating that *CsOxdC3* was not the primary gene responsible for OA degradation in this family. To verify the OA-degrading ability of the four *CsOxdC* genes, we analyzed the enzyme activity of the four gene deletion mutants (Figure 2E). The results revealed that the oxalate decarboxylase activities of the WT and Δ*CsOxdC3* strains were similar, while the enzyme activities of Δ*CsOxdC1*, Δ*CsOxdC2*, and Δ*CsOxdC4* were significantly decreased compared with the WT strain (Figure 2E). Taken together, these data indicated that *CsOxdC1*, *CsOxdC2*, and *CsOxdC4* played major roles in degrading OA in *C. siamense*, whereas *CsOxdC3* did not.

### 3.3 *CsOxdCs* positively regulates the vegetative growth, conidial morphology, conidial germination rate and appressorium formation rate of *Colletotrichum siamense* to different degrees

**Figure 3:**
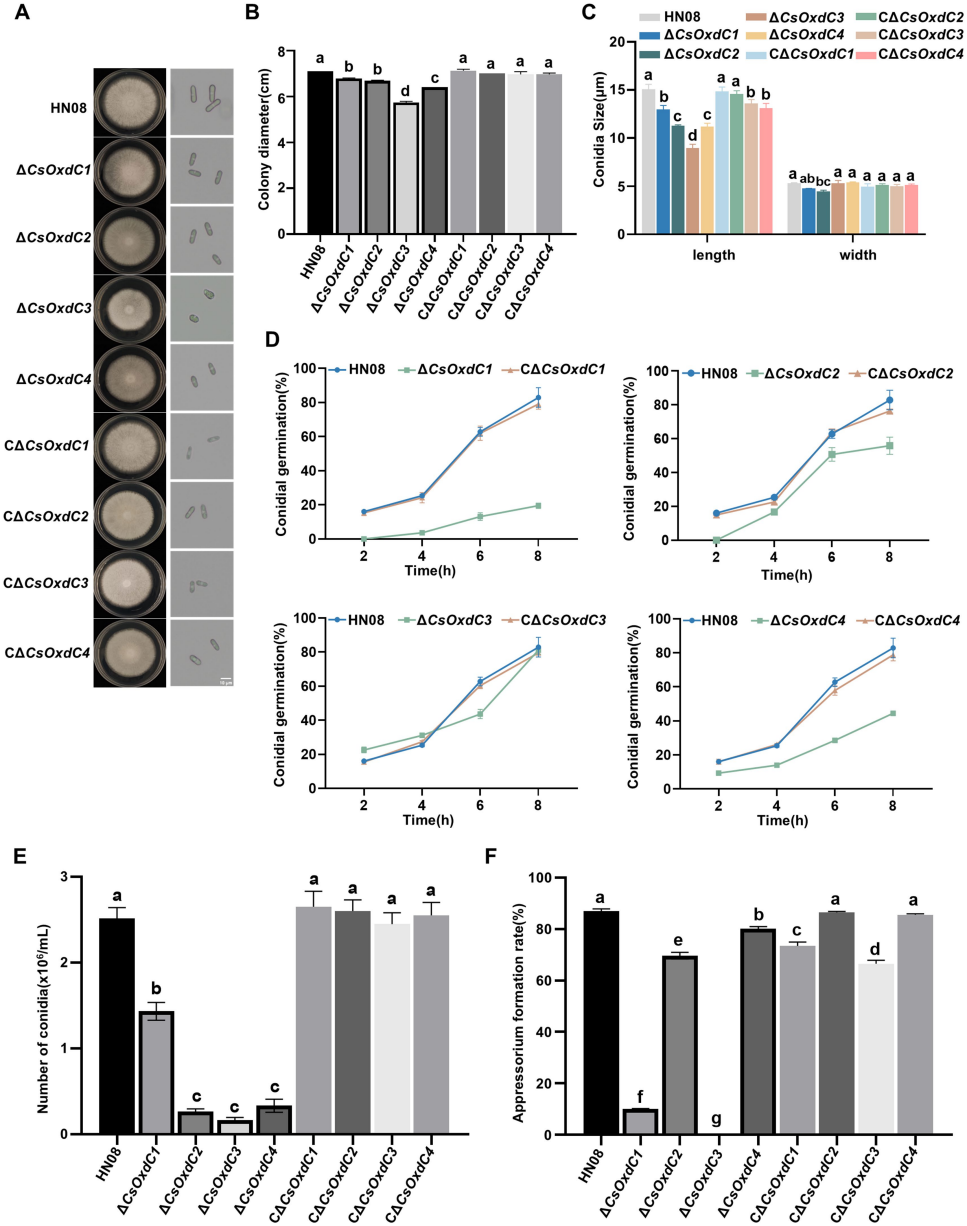
Effects of four *CsOxdC* genes on the growth and development of *C. siamense*. **(A)** Colony phenotype and conidia morphology of the tested strains cultured for 5 dpi. **(B)** Colony diameter of the tested strains. **(C)** Conidial size of the tested strains. **(D)** Conidial germination rates of the tested strains at 2, 4, 6 and 8 hpi. **(E)** Conidial number of the tested strain. **(F)** Appressorial formation rates of the tested strains. Different letters indicate significant differences (*p* < 0.05, according to one-way ANOVA and Duncan’s test), and the error bars represent the standard deviations.

To characterize the role of *CsOxdCs* in the vegetative growth and morphological development of *C. siamense*, we examined the radial growth and colony morphology of HN08, Δ*CsOxdCs*, and CΔ*CsOxdCs* cultured on minimal medium. Compared with the HN08 and complemented strains, the Δ*CsOxdCs* strains displayed a significant reduction in colony diameter (Figures 3A,B). Notably, Δ*CsOxdC3* exhibited the greatest reduction in colony diameter with a 19.30% reduction. The colony diameters of the Δ*CsOxdC1*, Δ*CsOxdC2* and Δ*CsOxdC4* mutants were reduced by 4.51, 5.92 and 9.86%, respectively (Figures 3A,B). These results indicated that the *CsOxdCs* family significantly influenced the colony growth of *C. siamense*, but the influence is relatively minor.

The conidial size, conidial germination rate, sporulation rate, and appressorium formation rates of the four mutants and the WT strain were also measured. The conidial sizes of the Δ*CsOxdC1*, Δ*CsOxdC2*, Δ*CsOxdC3*, and Δ*CsOxdC4* strains were (12.97 ± 0.34) × (4.77 ± 0.01) μm, (11.31 ± 0.05) × (4.46 ± 0.11) μm, (8.97 ± 0.32) × (5.31 ± 0.25) μm, and (11.21 ± 0.27) × (5.40 ± 0.04) μm, respectively, whereas that of the WT strain was (15.06 ± 0.42) × (5.3 ± 0.06) μm (Figures 3A,C). Data analyses revealed that the conidial lengths of the four gene deletion mutants were obviously shortened. The conidial germination rates of the Δ*CsOxdC1*, Δ*CsOxdC2*, and Δ*CsOxdC4* mutants were consistently lower than those of the WT from 2 to 8 hpi, whereas the rate of *∆CsOxdC3* was lower than that of the WT at 6 h but was similar to that of the WT at 8 hpi (Figure 3D). The sporulation of *∆CsOxdC1* (1.43 × 10^6^ conidia/mL, 43.25% decrease in conidiation), *∆CsOxdC2* (0.27 × 10^6^ conidia/mL, 89.29% decrease in conidiation), *∆CsOxdC3* (0.17 × 10^6^ conidia/mL, 93.25% decrease in conidiation), and *∆CsOxdC4* (0.33 × 10^6^ conidia/mL, 86.90% decrease in conidiation) was significantly lower than that of the WT strain (2.52 × 10^6^ conidia/mL) (Figure 3E). Additionally, the appressorium formation rates were also assessed, which were 87.06% at 12 hpi in WT compared with 10.07, 69.67%, 0, and 80.09% *∆CsOxdC1*, *∆CsOxdC2*, *∆CsOxdC3,* and *∆CsOxdC4*, respectively (Figure 3F). These results indicated that ∆*CsOxdC*s decreased the appressorium formation rate at 12 h, with ∆*CsOxdC1* and ∆*CsOxdC3* having the greatest impact. These data, coupled with the phenotypic recovery observed in the complementary strains (CΔ*CsOxdCs*), supported the conclusion that four *CsOxdCs* positively regulated the vegetative growth, conidial morphology, conidial germination rate and appressorium formation rate, but each gene affected them to different extents in *C. siamense*.

### 3.4 Diverse responses of *CsOxdC* genes in *Colletotrichum siamense* to Mn^2+^ and Cu^2+^ stresses

**Figure 4:**
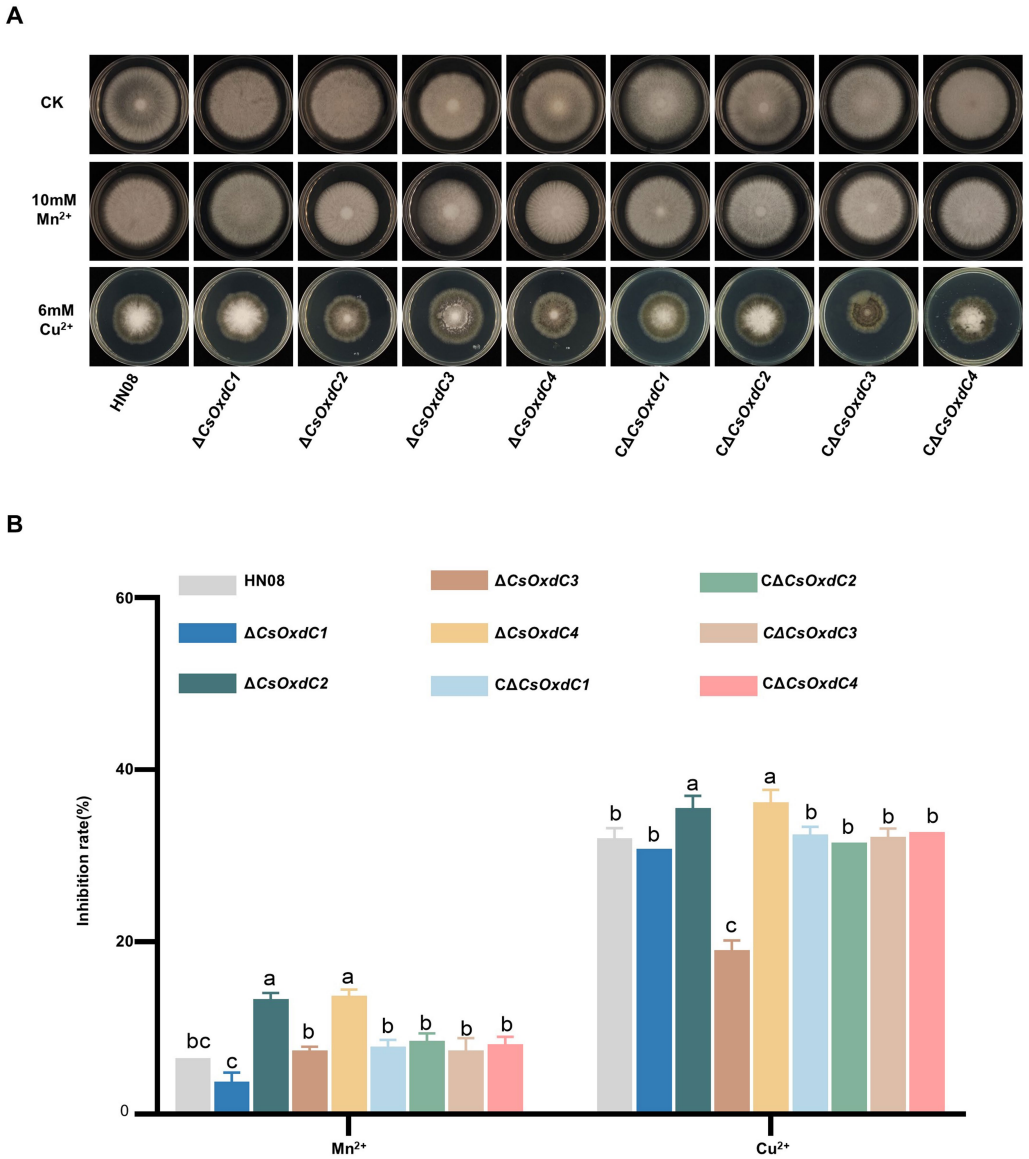
Comparison of responses to various stresses among WT (HN08), Δ*CsOxdCs* and CΔ*CsOxdCs*. **(A)** Mycelial growth of the tested strains on CM supplemented with 10 mM Mn^2+^ and 6 mM Cu^2+^ for 5 dpi. **(B)** Growth inhibition rates of the tested strains under different stresses. The growth inhibition rate is relative to the growth rate of each untreated control. Three repeats were performed. The error bars represent the standard deviations. Different letters indicate significant differences (*p* < 0.05, according to one-way ANOVA and Duncan’s test), and the error bars represent the standard deviations.

OxdCs act as manganese-containing polymerases and belong to the cupin superfamily (Mäkelä et al., 2010; Chowdhury et al., 2024), we investigated the contributions of the *CsOxdCs* gene to Mn^2+^ and Cu^2+^ stresses. The colony diameters of HN08, Δ*CsOxdCs*, and CΔ*CsOxdCs* on CM plates containing 10 mM Mn^2+^ and 6 mM Cu^2+^ were measured, and the inhibition rates were calculated (Figures 4A,B). Under Mn^2+^ stress, the inhibition rates of Δ*CsOxdC2* and Δ*CsOxdC4* were 13.37 and 13.77%, respectively, both of which were significantly greater than that in WT (6.53%). There were no significant differences between Δ*CsOxdC1* and HN08 or between Δ*CsOxdC3* and HN08 (Figure 4B). These findings indicated that the *CsOxdC2* and *CsOxdC4* genes were involved in the response to Mn^2+^ stress. Under Cu^2+^ stress, the inhibition rates of Δ*CsOxdC2* (35.56%) and Δ*CsOxdC4* (36.23%) were greater than that of WT (32.01%), whereas that of Δ*CsOxdC3* (19.04%) was lower than that of WT (Figure 4B). These findings indicated that *CsOxdC2* and *CsOxdC4* played positive roles in the regulation of *C. siamense* resistance to Cu^2+^ stress, whereas *CsOxdC3* had an opposite role. Taken together, these data suggested that four *CsOxdC* genes played diverse roles in the stress response of *C. siamense* to Mn^2+^ and Cu^2+^ stresses.

### 3.5 Different *CsOxdC* genes play various role in the regulation of fungicide sensitivity

**Figure 5:**
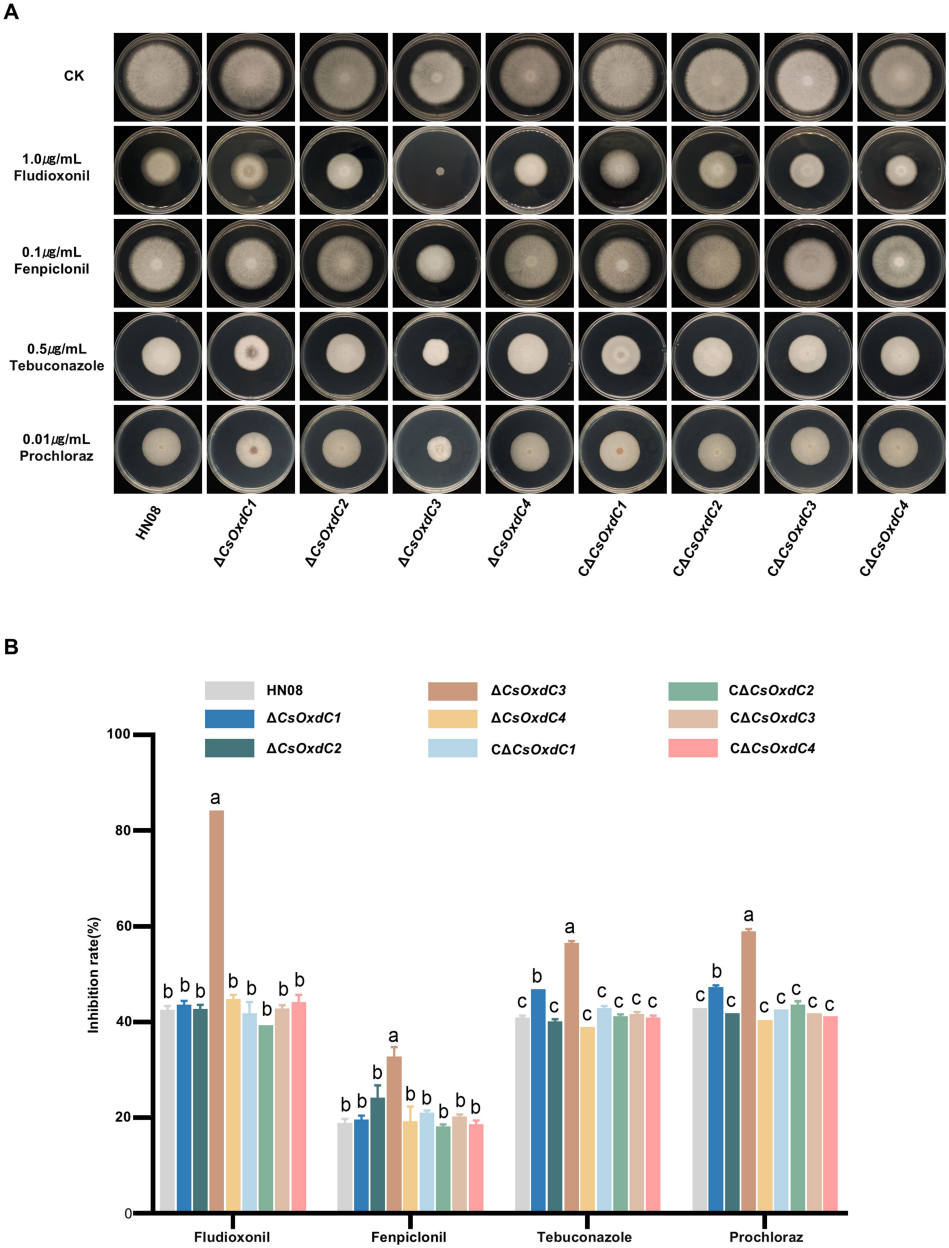
Comparison of fungicide sensitivity among WT (HN08), Δ*CsOxdCs*, CΔ*CsOxdCs*. **(A)** Mycelial growth of the tested strains on CM supplemented with 1.0 μg/mL dpi, 0.1 μg/mL Fenpiclonil, 0.5 μg/mL Tebuconazole and 0.01 μg/mL Prochloraz for 5 dpi. **(B)** Growth inhibition rates of the tested strains under different concentration of fungicides. The growth inhibition rate is relative to the growth rate of each untreated control. Three repeats were performed. The error bars represent the standard deviations. Different letters indicate significant differences (*p* < 0.05, according to one-way ANOVA and Duncan’s test), and the error bars represent the standard deviations.

Previous studies have shown that *CsOxdC3* is involved in regulating the sensitivity of *C. siamense* to pyrrole and azole fungicides (Lu et al., 2024). Here, we further evaluated whether other genes in this family played role in regulating the sensitivity of *C. siamense* to various fungicides. The growth inhibition rate of individual strains was assessed on CM plates supplemented with different fungicides, including fludioxonil, fenpiclonil, prochloraz, and tebuconazole (Figures 5A,B). The results showed that only deletion of the *CsOxdC3* gene, but not the other three genes, resulted in an increase in the growth inhibition rate under pyrrole fungicide (fludioxonil and fenpiclonil) stress. Under azole fungicide (prochloraz and tebuconazole) stress, the growth inhibition rates of both *∆CsOxdC3* and *∆CsOxdC1* were greater than those of WT. These results indicated that the *CsOxdC3* gene played a positive role in regulating the sensitivity of *C. siamense* to pyrrole and azole fungicides, which was consistent with previous research results (Lu et al., 2024). *CsOxdC1* also played a minor role in regulating the sensitivity of the strain to azole fungicides. These results suggested different *OxdC* genes played distinct roles in the regulation of fungicide sensitivity in *C. siamense*.

### 3.6 Both *CsOxdC3* and *CsOxdC2* are involved in *Colletotrichum siamense* virulence

**Figure 6:**
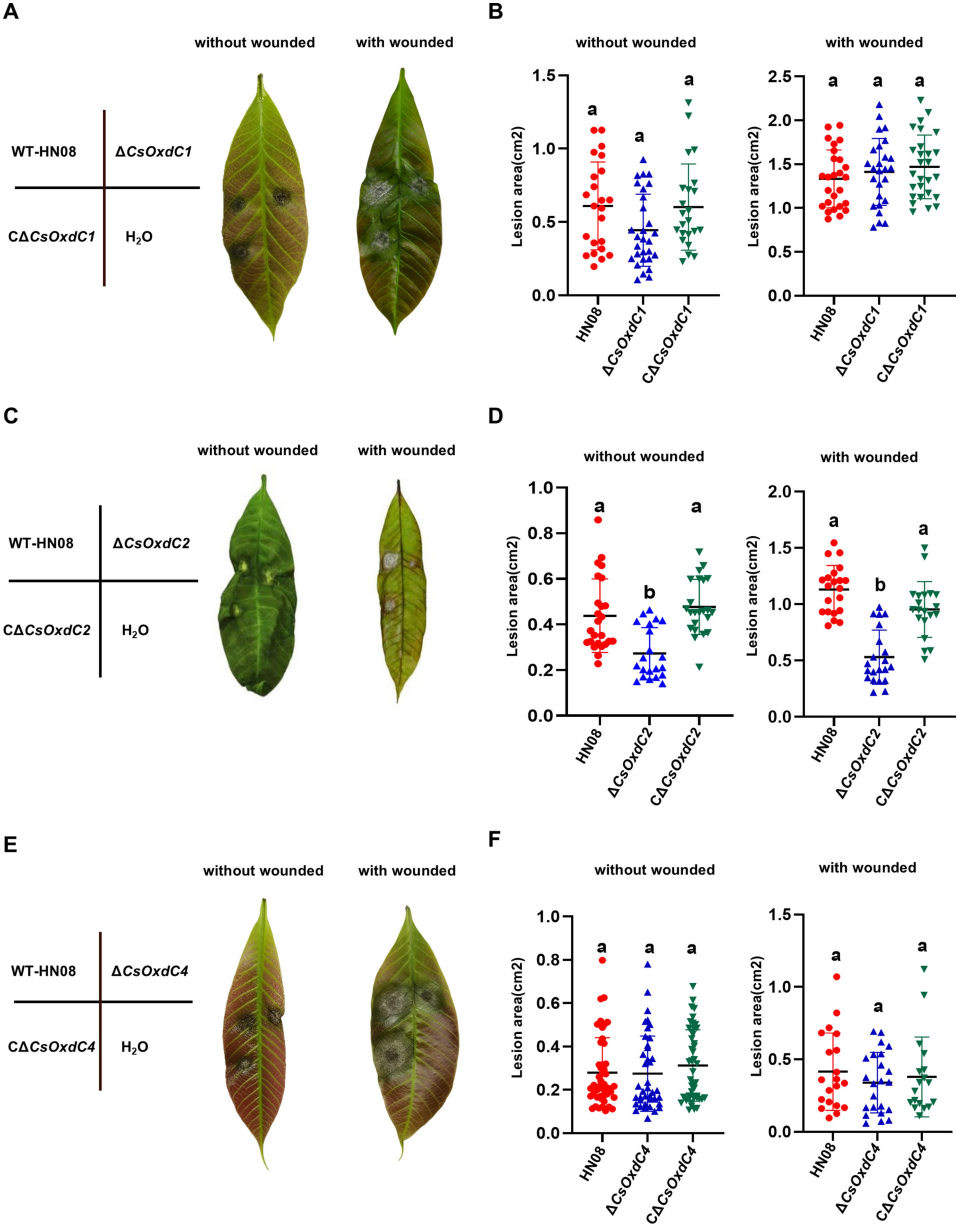
Virulence assays performed on rubber tree leaves. **(A,C,E)** Schematic diagram and symptoms of the virulence assays of the tested strains. The rubber tree leaves were inoculated with 10 μL of conidial suspension (1 × 10^5^ conidia/mL) of the tested strains with and without wounded. **(B,D,F)** Dot plot analysis of the lesion areas at 5 dpi. Different letters indicate significant differences (*p* < 0.05, according to one-way ANOVA and Duncan’s test), and the error bars represent the standard deviations.

Previous studies have shown that *∆CsOxdC3* attenuates the virulence of *C. siamense* (Lu et al., 2024). In this study, we tested the pathogenicity of three other gene deletion mutants. Conidial suspensions of the individual strains (10^5^ conidia/mL) were inoculated onto rubber tree leaves with and without wounding. The results revealed that the lesion areas caused by Δ*CsOxdC1* and Δ*CsOxdC4* were not significantly different from those caused by the WT strain or the complemented strains on both wounded and unwounded leaves. This finding indicated that deletion of the *CsOxdC1* and *CsOxdC4* genes did not significantly affect the pathogenicity of *C. siamense* (Figures 6A,B,E,F). However, the lesion areas infected by Δ*CsOxdC2*, HN08 and CΔ*CsOxdC2* on wounded leaves were 0.55 ± 0.28 cm^2^, 1.17 ± 0.36 cm^2^, and 0.91 ± 0.28 cm^2^, respectively. On unwounded leaves, the lesion areas were 0.31 ± 0.16 cm^2^, 0.41 ± 0.17 cm^2^, and 0.48 ± 0.12 cm^2^, respectively. Data analyses revealed that the lesion area caused by Δ*CsOxdC2* was significantly smaller than that caused by HN08 both on wounded and unwounded leaves, and the pathogenicity was restored to the WT level in the complemented strain CΔ*CsOxdC2* (Figures 6C,D). These results indicated that the *CsOxdC2* gene played an important role in the pathogenicity of *C. siamense* but not in *CsOxdC1* or *CsOxdC4*. The pathogenicity test of this study, along with previous studies, suggested that *CsOxdC2* and *CsOxdC3* in the *OxdCs* family were involved in the virulence of *C. siamense*.

## 4 Discussion

OxdC is a ubiquitous and common enzyme belonging to the cupin superfamily, which has a broad range of biochemical functions, including cell wall synthesis and oxidative processing (Azam et al., 2001; Chowdhury et al., 2024). Research on *OxdC* has focused mainly on its oxalate decarboxylase activity and potential applications, such as its ability to reduce calcium oxalate stones in medicine (Chakraborty et al., 2013; Lee et al., 2014; Sasikumar et al., 2014; Paul et al., 2018). In agriculture, most studies have focused mainly on heterologous expression of the *OxdC* gene to improve plant resistance to pathogens (Kesarwani et al., 2000; Dias et al., 2006; Walz et al., 2008; Qi et al., 2017; Pereira et al., 2022). Research on the biological function of *OxdCs* in microorganisms has been relatively limited to date. Previous studies on plant pathogenic fungi have shown that some *OxdCs* are involved in plant pathogenicity, especially necrotrophic fungi (Liang et al., 2015a). Little is known about the biological function of *OxdCs* in hemibiotrophic or biotrophic fungi. We previously reported that an OxdC-encoding gene, *CsOxdC3*, acts as an interactor of the mitogen-activated protein kinase CsPbs2, which is involved in morphogenesis, fungicide resistance and virulence in *C. siamense* (Lu et al., 2024). In this study, we systematically compared and analyzed the functions of all four *OxdCs* in morphogenesis, oxalate decarboxylase enzyme activity, stress regulation, fungicide resistance and virulence in *C. siamense* via the loss of single gene function method. We demonstrated that four individual genes exhibited distinct roles in the morphogenesis, enzyme activity, stress regulation, and pathogenicity of *C. siamense*, facilitating understanding of the biological functions of *OxdCs* in plant pathogenic fungi.

To survive, microorganisms usually evolve multiple genes to cope with the same biochemical processes to adapt to environmental conditions and stresses. These genes are redundant in certain functions and often exhibit differentiation. The presence of multiple *OxdCs* that degrade OA in fungi is a typical example. Previous studies have demonstrated the various functions of different *OxdCs* in fungi. For example, among three *OxdC* genes in *C. minitans*, two genes (*CmOxdc1* and *CmOxdc3*) are involved in OA degradation and the ability to parasitize the sclerotia of *S. sclerotiorum* (Zeng et al., 2014). In this study, we first systematically compared the oxalate decarboxylase activity of four *OxdC* genes in *C. siamense*. We found that three proteins, *CsOxdC1*, *CsOxdC2*, and *CsOxdC4*, possessed oxalate decarboxylase activity, whereas *CsOxdC3* exhibited the weakest oxalate degradation ability. Among these four proteins, the amino acid sequences of *CsOxdC3* and *CsOxdC4* were especially similar, with 76.41% homology, but their enzyme activities were quite different (Figure 2). This variation in enzymatic activity implied that different fungal *OxdC* genes might have diverse physiological and biological functions. Some of these genes might play specific roles in response to environmental conditions and stress factors.

In addition to their various enzymatic activities, different *OxdCs* have diverse functions in morphosporogenesis and stress regulation in *C. siamense*. Our findings demonstrated that *CsOxdCs* were involved in spore germination, sporulation, and appressorium formation, with each gene showing differential regulation. Among them, *CsOxdC3* played an important role in appressorium formation, which was abrogated in the gene deletion mutant (Figure 3F). In terms of their role in stress regulation, we also observed that four *OxdCs* played diverse roles in response to Mn^2+^ and Cu^2+^. *OxdC* is a manganese-containing polymerase (Mäkelä et al., 2010). Among these four *OxdCs*, *CsOxdC2* and *CsOxdC4* had positive roles in resistance to Mn^2+^ and Cu^2+^ stresses, and they exhibited relatively high oxalate decarboxylase activity. These results suggested that *CsOxdC2* and *CsOxdC4* served as major enzymes involved in the degradation of OA in *C. siamense*. With respect to fungicide resistance, our results demonstrated that *CsOxdC1* and *CsOxdC3* were involved in fungicide resistance regulation. *CsOxdC1* played a slightly positive role in *C. siamense* resistance to azole fungicides, and *CsOxdC3* played a significantly positive role in regulating the sensitivity of *C. siamense* to pyrrole and azole fungicides. We have previously reported that *CsOxdC3* interacts with the protein kinase CsPbs2, which is involved in fungicide resistance in *C. siamense* (Lu et al., 2024). We speculated that the differences in resistance of these OxdCs to various types of fungicides might be due to their different interacting proteins *in vivo*.

Some plant pathogenic fungi, especially necrotrophic fungi, utilize OA as a virulence factor or a nonspecific phytotoxin in interactions with plants (Cessna et al., 2000). Mutants of the fungus lacking the ability to produce OA show reduced pathogenicity (Rana et al., 2022). Acting as enzymes in the degradation of OA via decarboxylation, some *OxdCs* have also been reported to be associated with the pathogenicity of plant pathogenic fungi, such as *ss-odc2* in *S. sclerotiorum* (Liang et al., 2015a; Liang et al., 2015b). Among the four *OxdC* genes in *C. siamense*, *CsOxdC2* and *CsOxdC4* may be the major OA degradation enzymes, but *CsOxdC2* has a role in virulence; *CsOxdC4* shows no significant impact on pathogenicity. *CsOxdC3* deletion mutants also exhibited a significant reduction in virulence in previous studies (Lu et al., 2024), whereas *CsOxdC3* exhibited the weakest oxalate degradation ability in this study. *C. siamense* is a hemibiotrophic fungus, and OA may not be its major pathogenic factor. Because *CsOxdCs* are involved in spore germination, sporulation, and appressorium formation to different degrees, we speculated that the effects of *CsOxdC2* and *CsOxdC3* on the pathogenicity of *C. siamense* might involve not only OA but also a combination of multiple factors, including their effects on sporulation, spore germination and appressorium formation.

In summary, we systematically analyzed the biological functions of four oxalate decarboxylase genes in *C. siamense* in the present study, which was summarized in Supplementary Table S2. We revealed *CsOxdC1*, *CsOxdC2*, and *CsOxdC4* played major roles in degrading Oxalic acid in *C. siamense*, whereas *CsOxdC3* did not. Four *CsOxdCs* positively modulated morphosporogenesis, including vegetative growth, conidial size, conidial germination rate and the appressorium formation rate. These genes were also involved in stress homeostasis and fungicide resistance regulation to different extents. Furthermore, compared with WT, Δ*CsOxdC2* and Δ*CsOxdC3* exhibited significantly reduced virulence. In general, our study on the oxalate decarboxylase family in *C. siamense*, including aspects such as morphogenesis, stress homeostasis, fungicide resistance, and virulence, revealed that *CsOxdCs* have diverse functions and may operate through different pathways in *C. siamense*. These findings will help to elucidate the biological function of *OxdCs* in hemibiotrophic fungi.

## Data Availability

The original contributions presented in the study are included in the article/Supplementary material, further inquiries can be directed to the corresponding authors.
