# Supplementary material for: Functional diversification of oxalate decarboxylases in terms of enzymatic activity, morphosporogenesis, stress regulation and virulence in Colletotrichum siamense
Source: Front Microbiol. 2025 Feb 28;16:1547950. doi: 10.3389/fmicb.2025.1547950 (PMC11906461; doi:10.3389/fmicb.2025.1547950)

Figure S1. Schematic diagram and PCR verification of four single gene deletion mutants and their complemented strains of the *CsOxdC* genes in *C. siamense*.

A) Schematic diagram of *CsOxdCs* gene deletion and primers used to verify the gene replacement event. **B, C, D, E)** Represent PCR validation of Δ*CsOxdC1*, Δ*CsOxdC2*, Δ*CsOxdC3*, and Δ*CsOxdC4* and complemented strains, respectively. M: DNA DL5000 marker; Lanes 1-2 were products amplified by CsOxdCs-OUF/ ODR from Δ*CsOxdCs* (lane 1), WT (lane 2), respectively. Lanes 3-4 were products amplified by CsOxdCs-OUF/ ILV1-R from Δ*CsOxdCs* (lane 3), WT (lane 4), respectively. Lanes 5-6 were product amplified by CsOxdCs-F/R from Δ*CsOxdCs* (lane 5), WT (lane 6), respectively. Lanes 7-8 were product amplified by pBAR-GFP-F/R from CΔ*CsOxdCs* (lane 7), Δ*CsOxdCs* (lane 8), respectively.

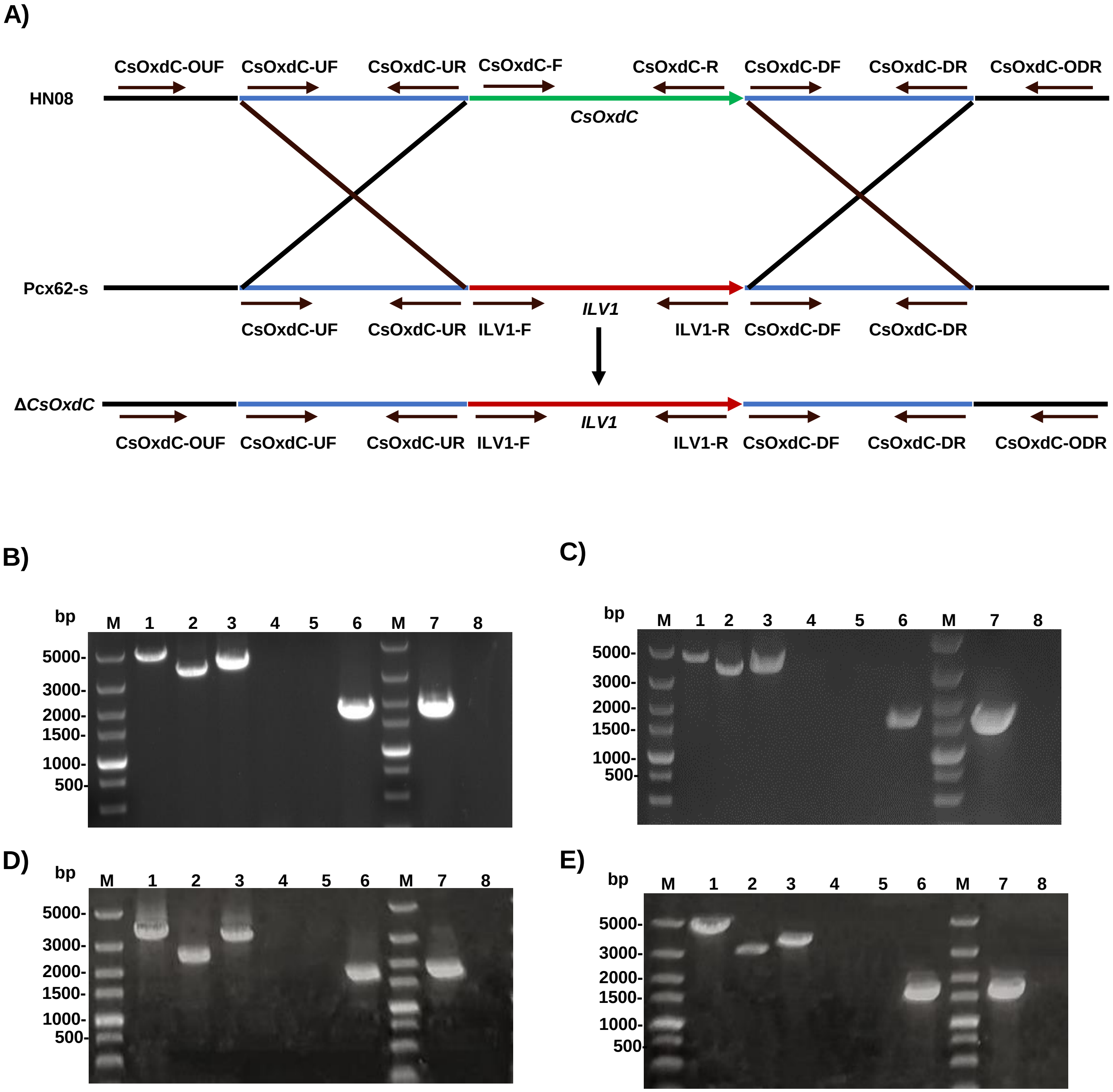

Figure S2. Tolerance of the mutants and the wild type of *C. siamense* to OA.
Colony size and medium color of the cultures of WT (HN08), mutants, and complementary strains on CM supplemented with bromophenol blue and with OA at 0, 3, 6, 12 and 24 mM. The letter of F and R stands for front and reverse of the plate, respectively. The yellow medium indicates low pH; blue indicates high pH.

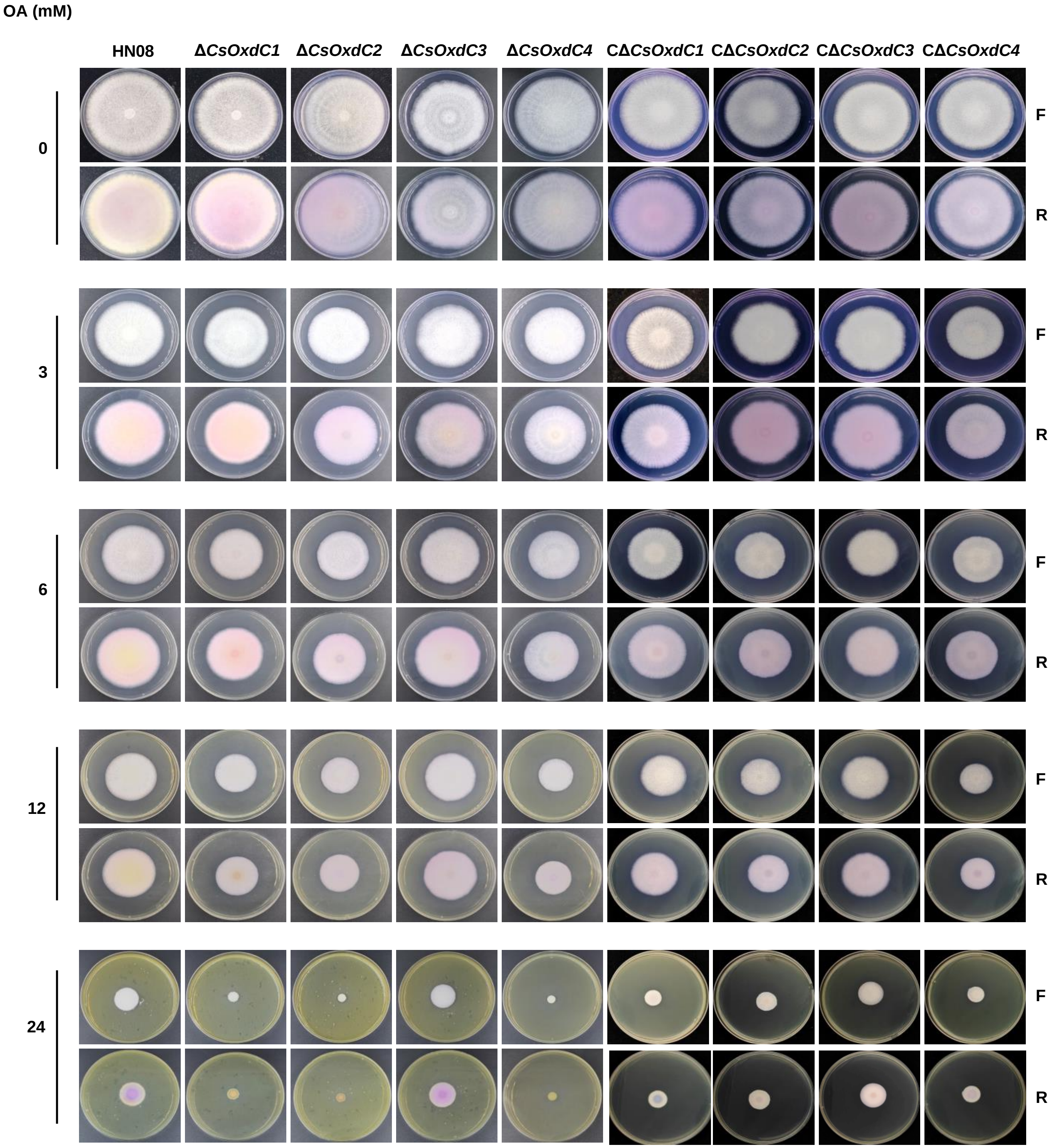

Supplement: Supplementary file 1 [file Data_Sheet_1.PDF]
