# Supplementary material for: Functional diversification of oxalate decarboxylases in terms of enzymatic activity, morphosporogenesis, stress regulation and virulence in Colletotrichum siamense
Source: Front Microbiol. 2025 Feb 28;16:1547950. doi: 10.3389/fmicb.2025.1547950 (PMC11906461; doi:10.3389/fmicb.2025.1547950)
Supplement: Supplementary file 2 [file Data_Sheet_2.PDF]

Table S1: List of primers used in the study

Primer name	Sequence (5'-3')
CsOxdC1-UF	GGTGATTATGGACACTGGTC
CsOxdC1-UR	AGATGTGGGGCACTGTGGCGTTGGCACGATGCTTGAATCTGACTGAAG
CsOxdC1-DF	TATTGCACGGGAATTGCATGCTCTCACTTTAGCGAATGGTGTCAATTG
CsOxdC1-DR	GACCAAGTACACTATCGCAC
CsOxdC1-F	ATGATCAGCCACGCAGCTTTC
CsOxdC1-R	CTAAGCCTTGAAGTTCTTACCAC
CsOxdC1-OUF	GTTCCTAACACAGGGTTGTAC
CsOxdC1-ODR	CGTTGTCAACGACATGTACC
pBAR-CsOxdC1-F	GACCTCGACTCTAGAGGATCCATGATCAGCCACGCAGCTTT
pBAR-CsOxdC1-R	GCCCTTGCTCACCATGGATCCAGCCTTGAAGTTCTTACCACTGCT
CsOxdC2-UF	GAGTCGTGAAGAGCACATGC
CsOxdC2-UR	AGATGTGGGGCACTGTGGCGTTGGCACGATGACCGGTACAACGAGTG
CsOxdC2-DF	TATTGCACGGGAATTGCATGCTCTCACACCATTCGGTGACGGTTGAAG
CsOxdC2-DR	GACATGCCATGAAGCACGAG
CsOxdC2-F	ATGAGACTCCTCCAAGCTTC
CsOxdC2-R	CTAGATCAGTATCTGCTTCTC
CsOxdC2-OUF	CTCGATCGAAGACGATGGTAG
CsOxdC2-ODR	GTGTCCTGCTAGACACCATC
pBAR-CsOxdC2-F	GACCTCGACTCTAGAGGATCCATGAGACTCCTCCAAGCTTCCC
pBAR-CsOxdC2-R	GCCCTTGCTCACCATGGATCCGATCAGTATCTGCTTCTCCTTCTTCA
CsOxdC3-UF	CACCAGACACCTCAACCTCAG
CsOxdC3-UR	AGATGTGGGGCACTGTGGCGTTGGCACGATGGGCGTGAAGTAGGAAGG
CsOxdC3-DF	TATTGCACGGGAATTGCATGCTCTCACGGGAGCACTGCCACAATGGAA
CsOxdC3-DR	GTTCAACCACACCGAGGTAG
CsOxdC3-F	ATGCCGGAGCCGTCCT
CsOxdC3-R	AAGTTCGTCGGTCTGAGTGC
CsOxdC3-OUF	GATAGCTGCGATTGCTCATG
CsOxdC3-ODR	GTGGTGATGGTGTTCACCTTC
pXY203-CsOxdC3-F	TTTCGTAGGAACCCAATCTTCAAAATGCCGGAGCCGTCCT
pXY203-CsOxdC3-R	TTCGAATTTAGCAGCAGCGGTTTCTTTAAGTTCGTCGGTCTGAGTGC
CsOxdC4-UF	GTAAGAGCATGTCGCAGTGTG
CsOxdC4-UR	GATGTGGGGCACTGTGGCGTTGGCACGGCTGGGCGCTGATGCTGAT
CsOxdC4-DF	GCACGGGAATTGCATGCTCTCACAGGAAAAGCAAAAGTGTCTTTG
CsOxdC4-DR	GACAAGGAGAATGGCAGCAG
CsOxdC4-F	ATGCAGCTCACCCAACCCA
CsOxdC4-R	CTACAATTCATCAGAAGCTCCG
CsOxdC4-OUF	CTGGGGCTAATCCGATTAGAG
CsOxdC4-ODR	CAGAATGGCAAGTCTGCGAAG
pBAR-CsOxdC4-F	GACCTCGACTCTAGAGGATCCATGCAGCTCACCCAACCCA
pBAR-CsOxdC4-R	GCCCTTGCTCACCATGGATCCCAACTCATCAGAAGCTCCGTCG
pBAR-GFP-F	CAAGCTTATCGATACCGTCG
pBAR-GFP-R	GAACTTGTGGCCGTTTACG
ILV1-F	GTGCCAACGCCACAGTGCCCCACA
ILV1-R	GTGAGAGCATGCAATTCCCGTGCAATA
Actin-F	TGGTATGGGCCAGAAGGA
Actin-R	GGACGGAAGGAGCGAACA
RT-CsOxdC1-F	GCTTTCCTTCTGGGGCTTCT
RT-CsOxdC1-R	TTTCCGGTAGGGCTTGTGAC
RT-CsOxdC2-F	GCTTCACTGGCACAAAGTGG
RT-CsOxdC2-R	GGCAACCCCTTCGGAAAGTA
RT-CsOxdC3-F	CACCCCTCGCTCCGTTATTT
RT-CsOxdC3-R	TTCTTGGGTTCCTGGGCAAG
RT-CsOxdC4-F	TGGATGGGAGAGACGCCTAA
RT-CsOxdC4-R	CCAGCCTTGACATCCGTCTT

Table S2: The summary of the enzyme activity and biological functions of four oxalate decarboxylase coding genes in *Colletotrichum siamense*.

Gene name	Enzyme activity	Role in growth and development					Respond to stress resistance				Pathogenicity
		Mycelial growth	Conidia length	Sporulation	Conidial Germination	Appressorium formation	Mn^{2+}	Cu^{2+}	Pyrrole fungicide	Azole fungicide	
CsOxdC1	++	+	+	+	+++	++	N	N	N	+	N
CsOxdC2	++	+	++	++	+	+	+	+	N	N	+
CsOxdC3	+	++	+++	++	+	+++	N	-	+	++	++
CsOxdC4	++	+	++	++	++	+	+	+	N	N	N

Note: +, means the gene play positive roles, the different number of symbols "+" represent different influence degree of the four genes in the same vertical column. "+++" indicates the highest degree of influence, "++" indicates the moderate influence, "+" indicates the least influence; -, means the gene play negative roles; N, means no impact.
